# Riboswitch Distribution in the Human Gut Microbiome Reveals Common Metabolite Pathways

**DOI:** 10.1021/acs.jpcb.4c00267

**Published:** 2024-04-24

**Authors:** Giulio Quarta, Tamar Schlick

**Affiliations:** † Department of Medicine, NYU Grossman School of Medicine, 450 East 29th St., Room 341, New York, New York 10016, United States; ‡ Department of Chemistry, New York University, 100 Washington Square East, Silver Building, New York, New York 10003, United States; § Courant Institute of Mathematical Sciences, New York University, 251 Mercer Street, New York, New York 10012, United States; ∥ New York University-East China Normal University Center for Computational Chemistry, New York University Shanghai, Shanghai 200122, China; ⊥ Simons Center for Computational Physical Chemistry, New York University, 24 Waverly Place, Silver Building, New York, New York 10003, United States

## Abstract

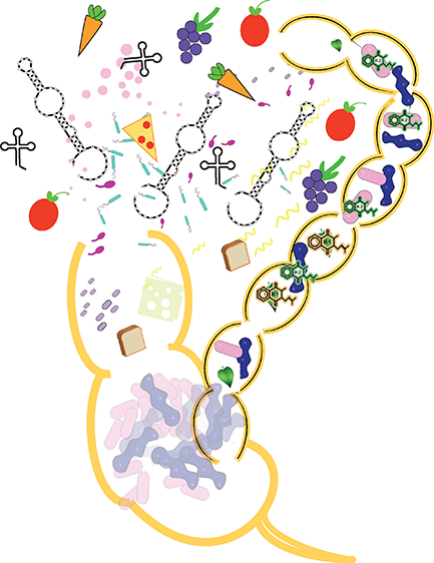

Riboswitches are widely distributed, conserved RNAs which regulate metabolite levels in bacterial cells through direct, noncovalent binding of their cognate metabolite. Various riboswitch families are highly enriched in gut bacteria, suggestive of a symbiotic relationship between the host and bacteria. Previous studies of the distribution of riboswitches have examined bacterial taxa broadly. Thus, the distribution of riboswitches associated with bacteria inhabiting the intestines of healthy individuals is not well understood. To address these questions, we survey the gut microbiome for riboswitches by including an international database of prokaryotic genomes from the gut samples. Using Infernal, a program that uses RNA-specific sequence and structural features, we survey this data set using existing riboswitch models. We identify 22 classes of riboswitches with vitamin cofactors making up the majority of riboswitch-associated pathways. Our finding is reproducible in other representative databases from the oral as well as the marine microbiomes, underscoring the importance of thiamine pyrophosphate, cobalamin, and flavin mononucleotide in gene regulation. Interestingly, riboswitches do not vary significantly across microbiome representatives from around the world despite major taxonomic differences; this suggests an underlying conservation. Further studies elucidating the role of bacterial riboswitches in the host metabolome are needed to illuminate the consequences of our finding.

## 1 Introduction

Gut microbes play an important role in human physiology, particularly through the production of small molecules.^1^ The vast number of bacteria present in the human intestine produce small molecules that affect host metabolism as well as other bacteria.^2^ Many of the bacterial metabolites are derived from host diets.^3^ Microbial metabolites enter into the host circulation,^4,5^ enteric and central nervous systems,^6^ and directly affect the physiology of the gastrointestinal tract.^7^

The exchange of metabolites from the gut microbiome with the host is an area of ongoing investigation.^8^ Many research groups have developed methods to quantify and classify gut microbial pathways, including computational databases^9^ and metabolomics pipelines.^2^ However, most bioactive small molecules in the gut are yet to be classified and functionally annotated due to the vast number of bacteria residing in the gut. As a first step, we focus on classifying and analyzing a highly prevalent class of functional RNAs in the gut microbiome called riboswitches.

**Figure 1:**
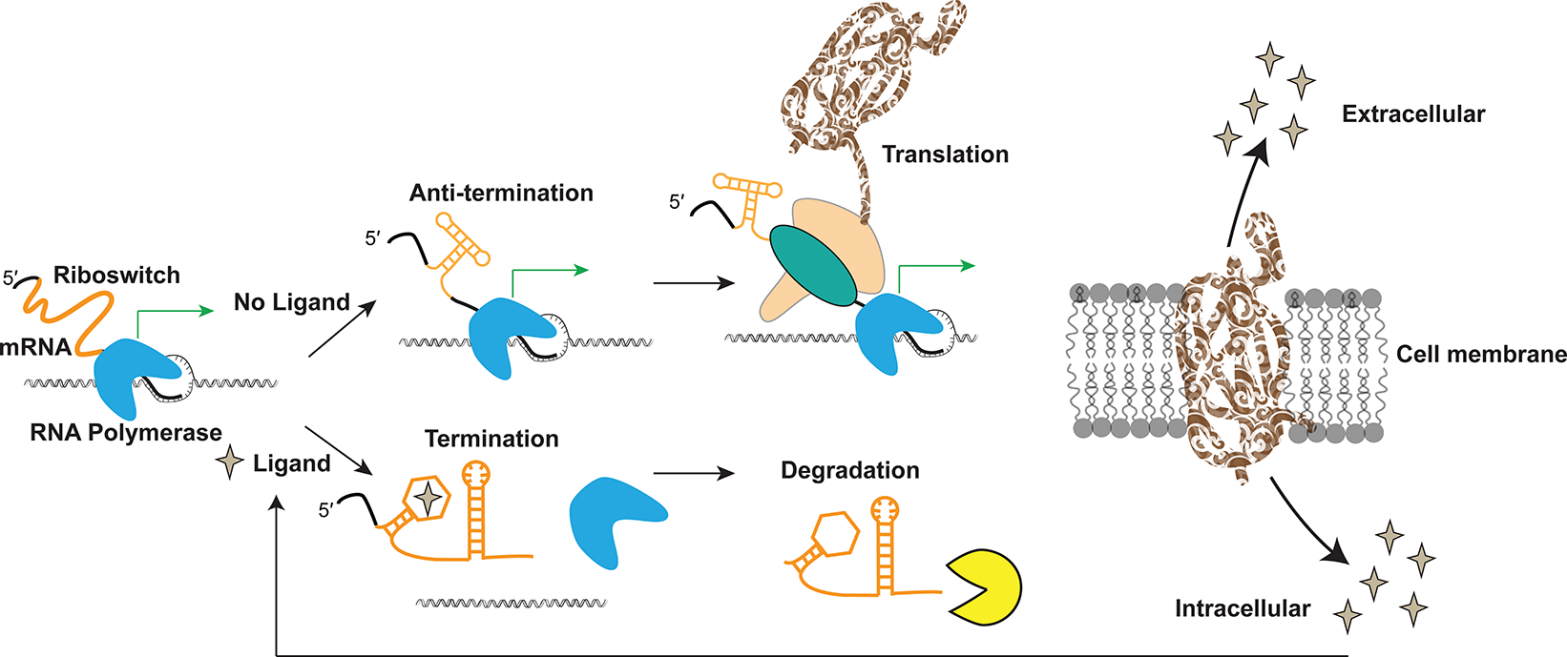
Canonical model of riboswitch regulation in bacteria. Riboswitches are typically found in the 5′ untranslated (UTR) regions of bacterial biosynthetic operons. In a transcription termination model, binding of an intracellular ligand (i.e., metabolite) leads to inhibition of transcription through a secondary structure formation of a terminator hairpin and eventual RNA degradation. When the cellular level of ligand is low (top panel), the riboswitch folds into an alternative conformation, leading to full transcription, translation, and protein expression; otherwise, transcription is suppressed (bottom panel). Most riboswitches are involved in feedback loops regulating the cellular concentration of their cognate ligand, thus regulating the cell metabolome.

Riboswitches are RNA regulatory control elements found throughout bacteria,^10^ plants,^11^ and fungi^12^ that perform direct, noncovalent binding to a specific intracellular metabolite.^10,13,14^ Riboswitches regulate the expression of specific genes and operons in bacteria (Figure 1) through a cascade of conformational switches: binding of a small molecule triggers a conformational change in the mRNA 5′ untranslated region (UTR), which in turn leads to transcription termination,^15^ RNA degradation,^16^ splicing,^17^ or translation inhibition.^18^ A reverse conformational change occurs when the product of the metabolic process is depleted.^19^

Thus, riboswitches regulate genes involved in the synthesis, import, or degradation of cognate metabolites.^20^ In this way, riboswitches help control the bacterial metabolome in hosts.^21^ Riboswitches are abundant in many gut microbes, though the extent to which riboswitches regulate levels of metabolites in the lumen of the gut remains unknown. Numerous taxonomic studies have been performed on the distribution and diversity of riboswitches across bacteria,^13,22,23^ but only a few studies aimed to understand riboswitch distribution in the human gut microbiome. Here, we use the Infernal package to survey the riboswitch classes present in the human gut microbiome and suggest that a core set of riboswitches are conserved across the gut microbiome, supporting the notion of a symbiotic relationship between gut bacteria and the host. Our study aims to determine if any group of riboswitch-associated pathways, out of the currently defined models, is enriched in the human gut microbiome.

## 2 Methods

The RNA family database (Rfam)^24^ was queried for riboswitches. Forty-one (*n* = 41) families were used in this study (Supporting Information Table S1), including five orphan classes, in which the metabolite is currently unknown. For metabolite-level classifications, we combined the following Rfam groups together:

B_12_: AdoCbl, AdoCbl_variant,
cobalaminc-di-GMP: class I
and IIPreQ1: class I, II, and
IIISAM: SAM, alpha, I–IV,
IV, VI, SMKmagnesium: ykoK
and Mg

We applied this collection of aptamer models to the UHGG data set,^25^ an international collection of prokaryotic genomes specifically from the gut, from samples across the world, fully annotated and assembled. In the version 2.0 data set, there is a set of 4728 genomes which constitute the representative data set. These species data can be found at https://www.ebi.ac.uk/metagenomics/browse#genomes. To correlate riboswitch locations to coding sequences (CDS), gene annotations were also collected for this data set, and the BEDtools window function was used to map the cmscan hits to the genome, using a 500nt window (-r 500) to find the nearest CDS and forcing directionality (-sm option).^26^ As comparators, the oral microbiome (*n* = 452 genomes), marine microbiome (*n* = 1496 genomes), and a matching random sample of mouse gut microbes^27^ (*n* = 4728 genomes) were used. For analysis of individual-level metagenomic data, we used a data set (*n* = 92) of gut metagenomes.^28^

For scanning and discovery of true aptamers in the genomic data sets, we applied cmscan (v1.1.5) from the Infernal suite.^29,30^ We used the trusted cutoff bit score threshold (—cut_ga) in the model to filter false positives. Statistical analysis was performed in R (v4.1) using the dplyr and forcats packages. For statistical comparisons between riboswitch and phylogenetic abundances, we used the 2-sample test for equality of proportions without continuity correction (Z-test).

To determine the pathway functions associated with each riboswitch class, we used the KEGG assignments derived from the UHGG data set. To match KEGG orthology to pathways, we input the cobalamin KEGG annotations into the KEGG “Map module”. https://www.genome.jp/kegg/ko.html.

## 3 Results

### 3.1 Gut-Specific Enrichment of Riboswitch-Associated Metabolic Pathways

To define the distribution of riboswitches across the gut microbiome, we use an international collection of fully annotated prokaryotic genomes representing 4616 bacteria and 28 archaea.^25^ To survey this genomic data set with Infernal,^29^ we apply 41 riboswitch RNA families from RFAM^24,30^ (Supporting Information Table S1). We identify 22 metabolite classes of riboswitches that are widely distributed across the gut microbiome with vitamin cofactors making up the majority of riboswitch-associated metabolic pathways (RAMPs) (Figure 1a). This finding is reproducible in representative sets from the human oral, mouse gut, and marine microbiomes. Thus, our survey suggests the importance of thiamine pyrophosphate (TPP, Vitamin B_1_), cobalamin (Vitamin B_12_), and flavin mononucleotide (FMN, Vitamin B_2_) in genetic regulation and central metabolism. RAMPs are found across taxa, with TPP most widely distributed across 14 distinct phyla (Figure 1b). Other widely distributed RAMPs in the gut include cobalamin (*n* = 12 phyla), FMN (*n* = 11), and S-adenosyl-methionine (*n* = 11).

We detect 36,844 riboswitches in the human gut microbiome, spanning over 10 million protein-coding sequences, which imply regulation of 0.3% of protein-coding sequences. However, the distribution of riboswitches is nonuniform, with the genomes of Fusobacteria, Synergistota, Firmicutes, and Proteobacteria particularly large (Figure 4). The organism *Peribacillus* simplex (UHGG: MGYG000000083) contains 61 independent examples of riboswitches within its genome, including S-adenosyl-methionine (*n* = 15), cobalamin (*n* = 9), and cyclic di-AMP (*n* = 7) aptamers (see Supporting Information File 1). The keystone member of the microbiota, *Bacteroides thetaiotamicron*, is associated with enrichment in cobalamin riboswitches (see Supporting Information File 1). These findings differ from the distribution of riboswitches across phylogenetic databases,^23^ suggesting that a subset of RAMPs are important for gut microbiome physiology.

**Figure 2:**
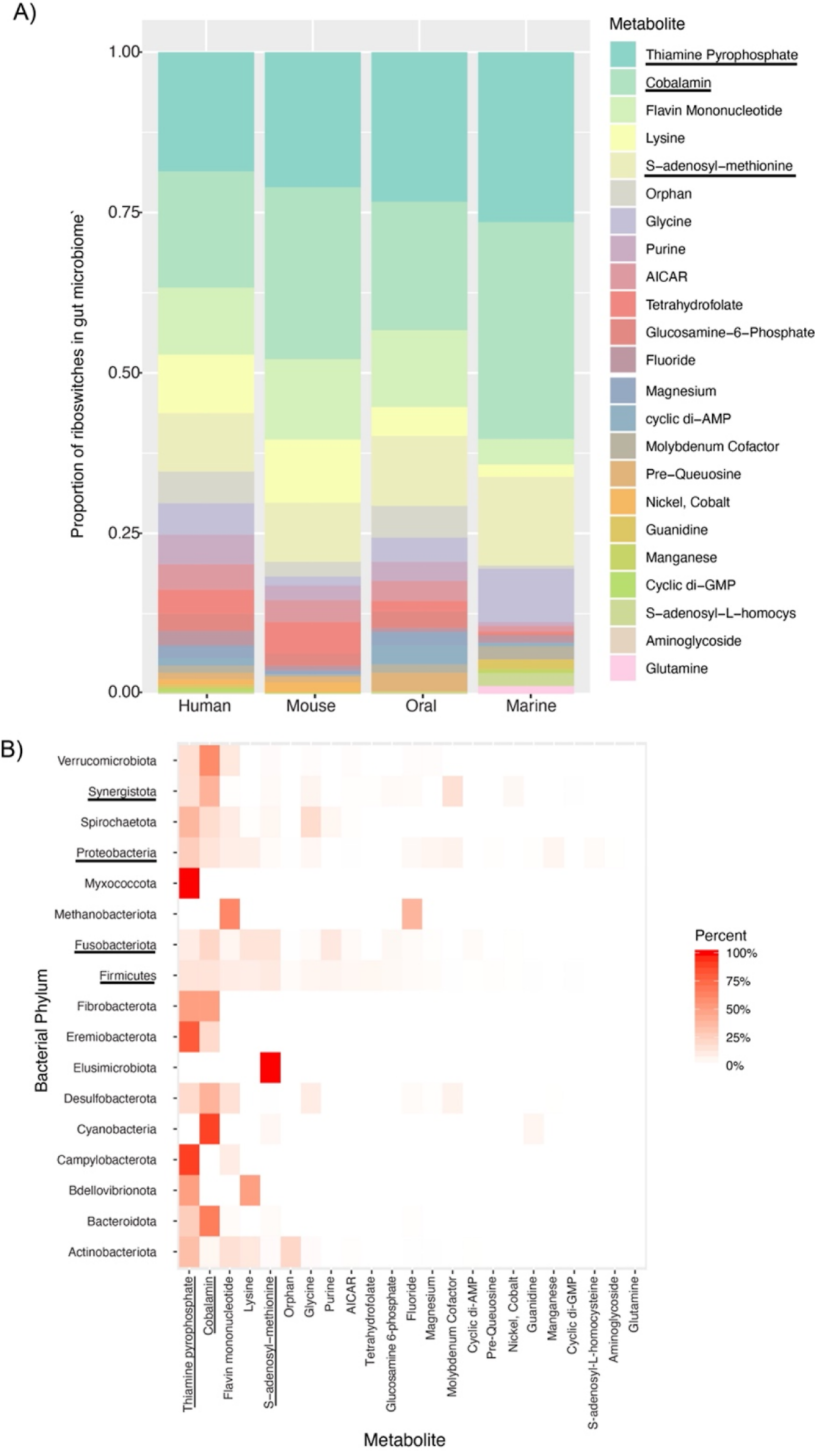
Comparison of riboswitch frequencies among microbiota and bacterial taxa of the gut microbiome. (A) Each class of riboswitch, according to a metabolite, is displayed with its proportion relative to the total number of riboswitches in each microbiome. Representative microbiome genomes were downloaded from UHGG (see Methods). The three species prevalent in many bacterial phyla in B are underlined. (B) Riboswitch frequency in the gut microbiome according to bacterial phylum. The four phyla of high frequency are underlined at left.

To search for organ-specific RAMPs, we compare distributions in the human gut against microbiome data sets from other organs. Human gut-enriched RAMPs include cyclic-di-AMP, cyclic-di-GMP, fluoride, magnesium, and manganese classes. Interestingly, the TPP, cobalamin, and FMN riboswitches are less abundant in human microbiomes in comparison to the other microbiomes studied despite being the most abundant classes (Figure 2a). In support of a role for riboswitch-mediated metabolic adaptation to ecological niche, we find that the glutamine riboswitch class is specific to the marine environment and not present in the gut or oral microbiomes.

**Figure 3:**
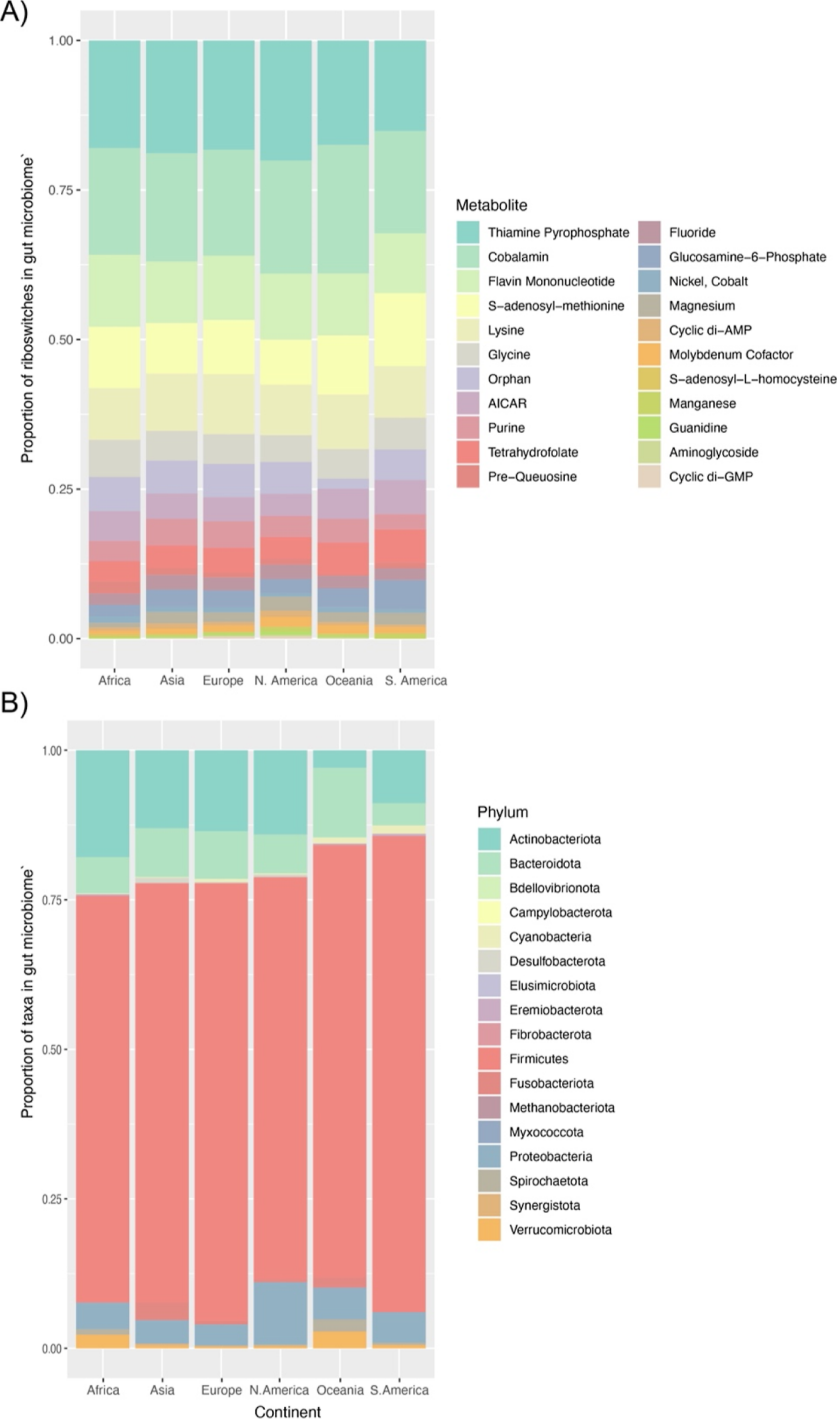
Average number of riboswitches in gut microbiome representatives from around the world. Panel (A) displays the relative proportion of each riboswitch class, per metabolite, from gut microbiome samples from around the world. Panel (B) displays the relative proportion of bacteria, according to Phylum, from the same gut microbiome samples from around the world.

We also compare the geologic distribution of specific riboswitch pathways to investigate the underlying physiology of human RAMPs (Figure 3). We wanted to examine whether riboswitches serve as markers of specific genetic pathways. We expected that dietary preferences and genetic differences among populations might influence the gut microbiome and the distribution of RAMPs. However, we find that riboswitches do not significantly differ between microbiome representatives from around the world (Figure 3a). Thus, no riboswitch class appears to be specific to any geographic region. Phylum-level comparison of taxa across groups reveals significant differences (Figure 3b). This suggests that RAMPs represent features of functional pathways conserved beyond the taxonomic classification.

### 3.2 Riboswitch Distribution in Individual Bacterial Genomes

**Figure 4:**
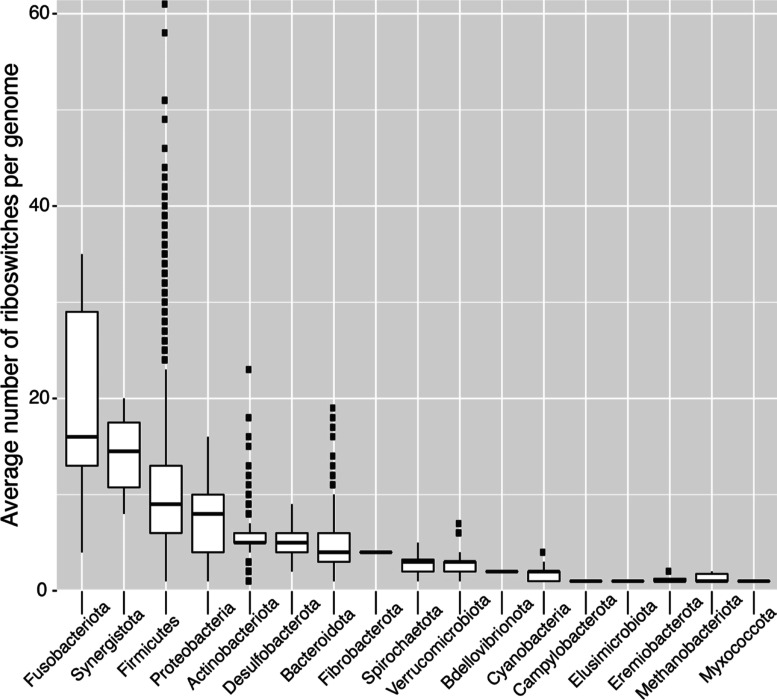
Average number of riboswitches per genome according to bacterial class.

The diverse collection of bacterial riboswitches across the gut microbiome presents metabolite-specific redundancy across individual genomes. Recent studies have revealed the conservation of metabolic pathways in gut bacteria beyond taxonomy (Figure 4). To survey coding regions associated with upstream riboswitches, we use the BEDtools suite^26^ to analyze the proteins and genetic pathways associated with RAMPs. We find that the vast majority of riboswitch-associated genes are involved in the transport of specific metabolites or the biosynthesis of aptamer-specific metabolites (Supporting Information Figure S1).

While many riboswitches can be involved in the transport or biosynthesis of metabolites, they also play diverse roles. All riboswitches associate with pathways beyond cognate ligand biosynthesis, ranging in function from central carbon metabolism, energy homeostasis, and antimicrobial resistance (Supporting Information Figure S1). These findings also support a role in the gut, playing multiple functional roles across bacterial taxa.

### 3.3 Interindividual Variability in Riboswitch Abundance in Metagenomic Samples

In shotgun metagenomic studies of the gut microbiome in which all microbial DNA is extracted and sequenced, applying functional and metabolic analyses engenders novel insights. We applied the Infernal program to individual patient samples from a recent data set analyzing the gut metagenome of healthy individuals.^28^ Similar to the analysis of the representative gut microbiome data set, the TPP, cobalamin, FMN, and SAM riboswitch classes are found in the highest abundance (Supporting Information File 1). Other classes of riboswitches, such as ZTP/AICAR and glucosamine-6-phosphate, are widely distributed but present at lower frequencies. Interestingly, we find a large variance in the number of copies of each riboswitch, suggesting a variation in the RAMP architecture. Other classes of riboswitches are neither widely distributed or abundant, such as SAH, guanidine, aminoglycoside, and manganese, consistent with their function in detoxification of certain chemicals in related organisms.^31^ Most riboswitch-associated metabolites can be found in both the serum and fecal metabolome,^2^ supporting a role of riboswitches as a direct effector on the host abundance of a number of molecules.

## 4 Discussion and Conclusions

We have surveyed a comprehensive gut microbial metagenomic data set for riboswitch aptamers and analyzed additional patient samples. Given that riboswitches are regulatory features of the gut metabolome, we expected them to be widely distributed, conserved across microbiomes, and specific to the metabolites that are important for stable microbial ecology. We have found that indeed specific classes of riboswitches, such as the vitamin cofactors thiamine pyrophosphate, flavin mononucleotide, and cobalamin, are abundant across taxa as well as geographies. Our findings agree with the critical role recognized for host-associated gut microbes for the production of vitamins in the health of animals.^32,33^ Because riboswitches control the transport of various metabolites and the biosynthesis of critical metabolites,^34^ their key role in the regulation of gene expression across taxa corresponds well to their abundance.

While human differences in diet and genetic factors contribute to diversity in the gut microbiome, our analysis did not find significant differences in any riboswitch architecture across geographic locations. On a global level, averaged over time, the RAMP architecture is conserved though we observe significant variations in riboswitch abundance on the individual level. This suggests that the riboswitch architecture is conserved broadly, though individual differences in diet and exposure to environmental factors explain riboswitch and, hence, metabolite differences in the gut. Further studies to elucidate the role of metabolite and riboswitch differences will help to expand on our findings.

Previous analyses of riboswitch classes in comprehensive surveys of all extant bacterial species have revealed trends similar to what we find here but also highlight key differences. In our analysis, we find that TPP and cobalamin aptamers are the top-ranking riboswitch classes across bacteria. However, in the gut microbiome, the abundance of FMN and lysine aptamers ranks higher than outside the gut. The reasons for these differences possibly relate to the requirement of the host to use these metabolites from the gut: specific transporters in the intestines exist for TPP, cobalamin, FMN, and lysine.^33,35^ In addition, we found that cyclic-di-AMP and cyclic-di-GMP riboswitches are over-represented in the gut microbiome, likely related to the roles of cyclic dinucleotides in biofilm formation and potent immune regulation.^36^

Our survey has likely produced a conservative estimate of the number of riboswitches distributed across the gut microbiome data set. We use a threshold in Infernal that specifically filters false-positive hits to a high degree, as certain aptamers can vary in metabolic binding with minimal sequence and structural variation. Since prokaryotic genes are organized into multiple linked genes that form an operon, the effect of riboswitch regulation is likely much higher than the 0.3% of genes found to have riboswitches associated with their regulation. Orphan riboswitch discovery is an area of active research that will inevitably broaden our understanding of how riboswitches regulate genes across taxa. While our study uses representative genomes, future work could consider the analysis of riboswitch aptamers in metagenomic or transcriptomic data sets. In addition, the biochemical significance and consequences of our findings could be further explored by experiments that measure the metabolite of interest in the host, fecal microbiome, in tandem with quantitative expression levels of the riboswitch pathway of interest.

## Data Availability

The data used in this study was retrieved from public repositories. The species data can be found at: https://www.ebi.ac.uk/metagenomics/browse#genomes. The individual metagenomic data can be found at: https://ftp.cngb.org/pub/gigadb/pub/10.5524/100001_101000/100548/METAGENOMIC_ANALYSIS_FILES/. All scripts used to process and analyze the data can be found in Supporting Information File 2.

## Supporting Information Available

Number and distribution of riboswitches in *Peribacillus* simplex and *B. thetaiotamicron* and in individual patient samples (XLSX)
Association of the cobalamin (B_12_) riboswitch with specific genetic pathways and Rfam models used in this study (PDF)
Scripts used in the analysis (PDF)
