# Supplementary material for: Riboswitch Distribution in the Human Gut Microbiome Reveals Common Metabolite Pathways
Source: J Phys Chem B. 2024 Apr 24;128(18):4336–43. doi: 10.1021/acs.jpcb.4c00267 (PMC11089507; doi:10.1021/acs.jpcb.4c00267)
Supplement: Supplementary file 2 — jp4c00267_si_002.pdf [file jp4c00267_si_002.pdf]

Riboswitch Distribution in the Human Gut Microbiome Reveals Common Metabolite Pathways

Giulio Quarta[1,*] and Tamar Schlick[2,3,4,*]

[1]Department of Medicine, 450 East 29th St., Room 341, NYU Grossman School of Medicine, New York, NY 10016, USA

[2]Department of Chemistry, 100 Washington Square East, Silver Building, New York University, New York, NY 10003, USA

[3]Courant Institute of Mathematical Sciences, New York University, 251 Mercer St., New York, NY 10012, USA

[4]New York University-East China Normal University Center for Computational Chemistry, New York University Shanghai, Shanghai 200122, China

[5]Simons Center for Computational Physical Chemistry, 24 Waverly Place, Silver Building, New York University, New York, NY 10003, USA

*To whom correspondence should be addressed: giulio.quarta@nyulangone.org, schlick@nyu.edu

Supplementary Figures and Tables:

Supplementary Figure 1 - Association of the cobalamin (B_{12}) riboswitch with specific genetic pathways.
Supplementary Table 1 – Rfam models used in this study.

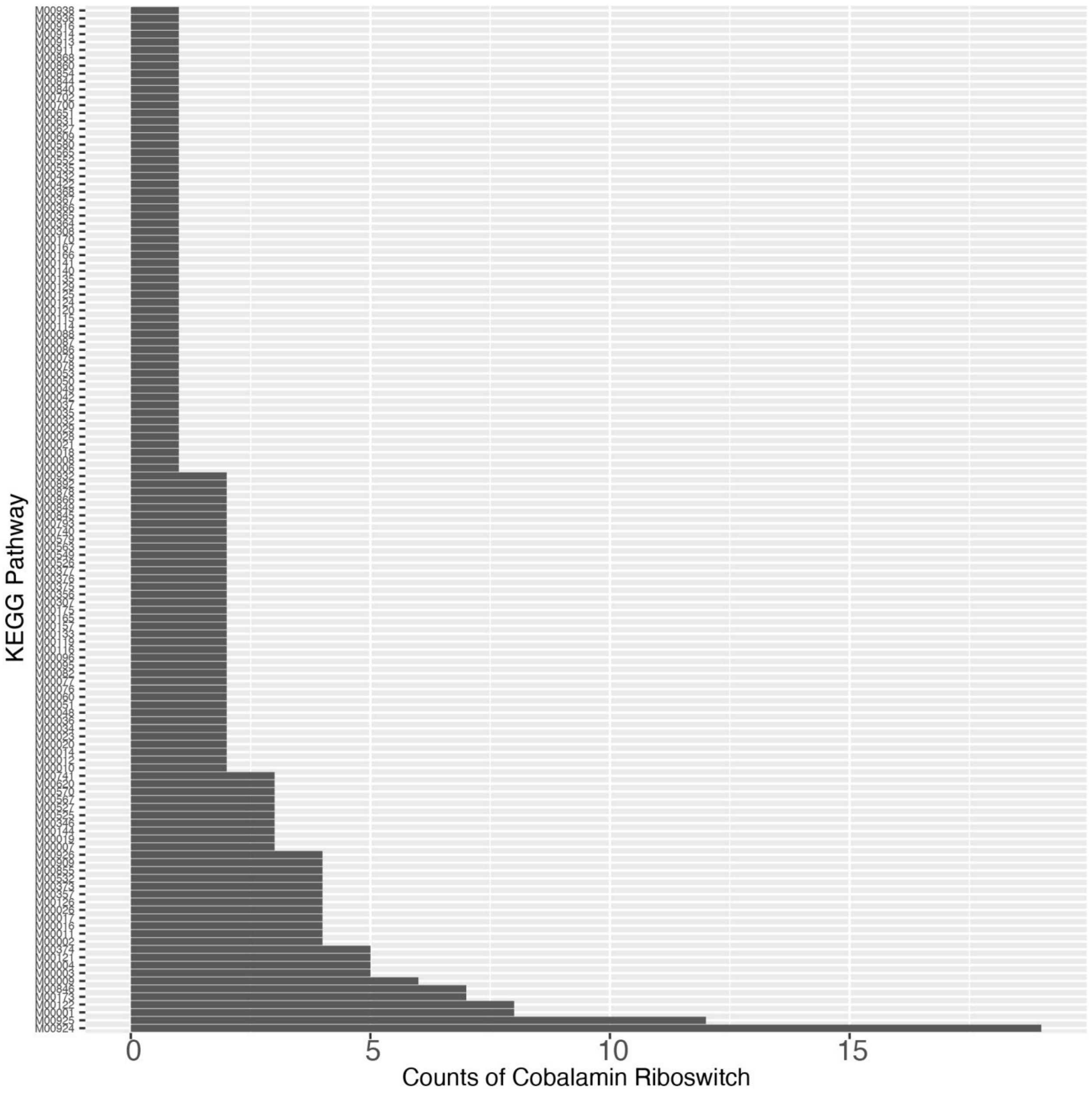

Supplementary Figure 2 - Association of the cobalamin (B_{12}) riboswitch with specific genetic pathways. Each line represents a Cobalamin (B_{12}) riboswitch associated with a separate KEGG pathway (https://www.genome.jp/kegg/pathway.html), sorted according to the number of hits in the human gut microbiome dataset.

Supplementary Table 1

RF00059 TPP Riboswitch	RF01051 Cyclic di-GMP-I riboswitch
RF00167 Purine Riboswitch	RF03058 sul1 RNA. orphan
RF00050 FMN Riboswitch	RF01727 SAM/SAH riboswitch
RF03071 DUF1646 RNA. Orphan	RF01057 S-adenosyl-L-homocysteine riboswitch
RF01689 AdoCbl variant RNA. cobalamin	RF00168 Lysine riboswitch
RF00380 M-box riboswitch (ykoK leader)	RF00504 Glycine riboswitch
RF01055 Moco bbc(molybdenum cofactor) riboswitch	RF00634 S-adenosyl methionine (SAM) IV riboswitch
RF01750 ZMP/ZTP riboswitch	RF00521 SAM riboswitch (alpha-proteobacteria)
RF03057 nhaA-I RNA. orphan	RF00522 PreQ1 riboswitch
RF01734 Fluoride riboswitch	RF00080 yybP-ykoY manganese riboswitch
RF01725 SAM-I/IV variant riboswitch. SAM	RF02680 PreQ1-III riboswitch
RF00174 Cobalamin riboswitch	RF01767 SMK box translational riboswitch (SAM-III)
RF00162 SAM riboswitch (S box leader)	RF00234 glmS glucosamine-6-phosphate activated ribozyme
RF03072 raiA RNA	RF01054 preQ1-II (pre queuosine) riboswitch
RF01739 Glutamine riboswitch	RF01482 AdoCbl riboswitch
RF00379 ydaO/yuaA leader. cyclic di-AMP	RF02885 SAM-VI riboswitch
RF01831 THF riboswitch	RF01826 SAM-V riboswitch
RF00442 Guanidine-I riboswitch	RF02912 putative aminoglycoside riboswitch / attI site
RF02683 NiCo riboswitch	RF01056 Magnesium Sensor
RF01786 Cyclic di-GMP-II riboswitch	RF01510 M. florum riboswitch
	RF01764 YJDF
