# Supplementary material for: Riboswitch Distribution in the Human Gut Microbiome Reveals Common Metabolite Pathways
Source: J Phys Chem B. 2024 Apr 24;128(18):4336–43. doi: 10.1021/acs.jpcb.4c00267 (PMC11089507; doi:10.1021/acs.jpcb.4c00267)
Supplement: Supplementary file 3 — jp4c00267_si_003.pdf [file jp4c00267_si_003.pdf]

Riboswitch Distribution in the Human Gut Microbiome Reveals Common Metabolite Pathways

Giulio Quarta[1,*] and Tamar Schlick[2,3,4,*]

[1]Department of Medicine, 450 East 29th St., Room 341, NYU Grossman School of Medicine, New York, NY 10016, USA

[2]Department of Chemistry, 100 Washington Square East, Silver Building, New York University, New York, NY 10003, USA

[3]Courant Institute of Mathematical Sciences, New York University, 251 Mercer St., New York, NY 10012, USA

[4]New York University-East China Normal University Center for Computational Chemistry, New York University Shanghai, Shanghai 200122, China

[5]Simons Center for Computational Physical Chemistry, 24 Waverly Place, Silver Building, New York University, New York, NY 10003, USA

*To whom correspondence should be addressed: giulio.quarta@nyulangone.org, schlick@nyu.edu

Supplementary Data file 2

Hypothesis:

analyze the distribution of riboswitches across the gut microbiome

Rfam queried for all riboswitches. The following list of 41 families found, with one control (tmRNA) included:

1. RF00059 TPP Riboswitch
2. RF00167 Purine Riboswitch
3. RF00050 FMN Riboswitch
4. RF03071 DUF1646 RNA. Orphan
5. RF01689 AdoCbl variant RNA. cobalamin
6. RF00380 M-box riboswitch (ykoK leader)
7. RF01055 Moco bbc(molybdenum cofactor) riboswitch
8. RF01750 ZMP/ZTP riboswitch
9. RF03057 nhaA-I RNA. orphan
10. RF01734 Fluoride riboswitch
11. RF01725 SAM-I/IV variant riboswitch. SAM
12. RF00174 Cobalamin riboswitch
13. RF00162 SAM riboswitch (S box leader)
14. RF03072 raiA RNA
15. RF01739 Glutamine riboswitch
16. RF00379 ydaO/yuaA leader. cyclic di-AMP
17. RF01831 THF riboswitch
18. RF00442 Guanidine-I riboswitch
19. RF02683 NiCo riboswitch
20. RF01786 Cyclic di-GMP-II riboswitch
21. RF01051 Cyclic di-GMP-I riboswitch
22. RF03058 sul1 RNA. orphan

23. RF01727 SAM/SAH riboswitch
24. RF01057 S-adenosyl-L-homocysteine riboswitch
25. RF00168 Lysine riboswitch
26. RF00504 Glycine riboswitch
27. RF00634 S-adenosyl methionine (SAM) IV riboswitch
28. RF00521 SAM riboswitch (alpha-proteobacteria)
29. RF00522 PreQ1 riboswitch
30. RF00080 yybP-ykoY manganese riboswitch
31. RF02680 PreQ1-III riboswitch
32. RF01767 SMK box translational riboswitch (SAM-III)
33. RF00234 glmS glucosamine-6-phosphate activated ribozyme
34. RF01054 preQ1-II (pre queuosine) riboswitch
35. RF01482 AdoCbl riboswitch
36. RF02885 SAM-VI riboswitch
37. RF01826 SAM-V riboswitch
38. RF02912 putative aminoglycoside riboswitch / attI site
39. RF01056 Magnesium Sensor
40. RF01510 M. florum riboswitch
41. RF01764 YJDF
42. RF00023 tmRNA

Dataset UHGG Gut Microbiome:
https://www.nature.com/articles/s41587-020-0603-3
This is an international collection of prokaryotic genomes specifically from the gut from samples across the world, fully annotated and assembled.

To browse the dataset:
https://www.ebi.ac.uk/metagenomics/browse#genomes

In this dataset there is a set of 4,644 gut species (4,728 in v2.0) which represent the representative dataset. These species data can be found at:
http://ftp.ebi.ac.uk/pub/databases/metagenomics/mgnify_genomes/human-gut/v2.0/species_catalogue/

Dataset Control Oral Microbiome:
This is a good comparator to the gut microbiome. It is also human, annotated the same, and representative of a large sample size.
In this dataset there is a set of species which represent the representative dataset of oral taxa. These species data can be found at:
http://ftp.ebi.ac.uk/pub/databases/metagenomics/mgnify_genomes/human-oral/v1.0/species_catalogue/

Dataset Control Marine Microbiome:
This is a good comparator to the gut microbiome.
In this dataset there is a set of species which represent the representative dataset of oral taxa. These species data can be found at:
http://ftp.ebi.ac.uk/pub/databases/metagenomics/mgnify_genomes/marine/v1.0/species_catalogue/

Dataset Mouse Gut Microbiome

Go to https://github.com/BenBeresfordJones/MGBC
Downloaded: 26,640 high-qual, non-redundant genomes
Filename: MGBC-hqnr_26640.tar.gz

Dataset Human gut metagenomic samples
This is a good sample of the gut microbiome.
In this dataset there are gut metagenomes which are not fully assembled. These data can be found at:
https://ftp.cngb.org/pub/gigadb/pub/10.5524/100001_101000/100548/METAGENOMIC_ANALYSIS_FILES/Assembled_Contigs/

Scanning and searching for Riboswitches in the representative genomes:
Step 1: create an CM database flatfile

```
#Download all Covariance Models above:
cat RF0*cm > riboswitch_fam.cm
cmpress riboswitch_fam.cm
##this creates a compressed CM of all the riboswitches
```

Step 2: Script to search the genomes for the riboswitches

```
cmscan -o tmp2 --cpu 12 --noali --cut_tc  --tblout *.tblout
riboswitch_fam.cm *.input_file | sed 's/.fna.tblout/.tblout/' >
Rfam_search
```

Analysis and distribution of riboswitches in gut microbiome:
Step 1: cleanup the *tblout files.

First, in the genomes-all_metadata.tsv file convert “South America” to “South_America” and “North America” to “North_America” and “not provided” to “NA”.
Remove the # lines, remove extra words from the ends of lines, paste in the taxonomy information from genomes_all-metadata.csv as well as the continent of origin. Then concatenate all the files together.

Statistical analysis in R

```
library(RColorBrewer)
library(ggplot2)
library(dplyr)
library(forcats)

a<-read.table("Marine_Rfam_concat_cleaned.tblout")
#plot(table(a[,1])) #plot counts off all riboswitches across all
a[which(a[,2]=="RF00059"),27]<-"Thiamine Pyrophosphate"
a[which(a[,2]=="RF00167"),27]<-"Purine"
a[which(a[,2]=="RF00050"),27]<-"Flavin Mononucleotide"
a[which(a[,2]=="RF03071"),27]<-"Orphan"
a[which(a[,2]=="RF01689"),27]<-"Cobalamin"
a[which(a[,2]=="RF00380"),27]<-"Magnesium"
a[which(a[,2]=="RF01055"),27]<-"Molybdenum Cofactor"
a[which(a[,2]=="RF01750"),27]<-"AICAR"
a[which(a[,2]=="RF03057"),27]<-"Orphan"
a[which(a[,2]=="RF01734"),27]<-"Fluoride"
a[which(a[,2]=="RF01725"),27]<-"S-adenosyl-methionine"
a[which(a[,2]=="RF00174"),27]<-"Cobalamin"
a[which(a[,2]=="RF00162"),27]<-"S-adenosyl-methionine"
a[which(a[,2]=="RF03072"),27]<-"Orphan"
a[which(a[,2]=="RF01739"),27]<-"Glutamine"
a[which(a[,2]=="RF00379"),27]<-"cyclic di-AMP"
a[which(a[,2]=="RF01831"),27]<-"Tetrahydrofolate"
a[which(a[,2]=="RF00442"),27]<-"Guanidine"
a[which(a[,2]=="RF02683"),27]<-"Nickel, Cobalt"
a[which(a[,2]=="RF01786"),27]<-"Cyclic di-GMP"
a[which(a[,2]=="RF01051"),27]<-"Cyclic di-GMP"
a[which(a[,2]=="RF03058"),27]<-"Orphan"
a[which(a[,2]=="RF01727"),27]<-"S-adenosyl-methionine"
a[which(a[,2]=="RF01057"),27]<-"S-adenosyl-L-homocysteine"
a[which(a[,2]=="RF00168"),27]<-"Lysine"
a[which(a[,2]=="RF00504"),27]<-"Glycine"
a[which(a[,2]=="RF00634"),27]<-"S-adenosyl-methionine"
a[which(a[,2]=="RF00521"),27]<-"S-adenosyl-methionine"
a[which(a[,2]=="RF00522"),27]<-"Pre-Queuosine"
a[which(a[,2]=="RF00080"),27]<-"Manganese"
a[which(a[,2]=="RF02680"),27]<-"Pre-Queuosine"
a[which(a[,2]=="RF01767"),27]<-"S-adenosyl-methionine"
a[which(a[,2]=="RF00234"),27]<-"Glucosamine-6-Phosphate"
a[which(a[,2]=="RF01054"),27]<-"Pre-Queuosine"
a[which(a[,2]=="RF01482"),27]<-"Cobalamin"
a[which(a[,2]=="RF02885"),27]<-"S-adenosyl-methionine"
a[which(a[,2]=="RF01826"),27]<-"S-adenosyl-methionine"
```

```
a[which(a[,2]=="RF02912"),27]<-"Aminoglycoside"
a[which(a[,2]=="RF01056"),27]<-"Magnesium"
a[which(a[,2]=="RF01510"),27]<-"Purine"
a[which(a[,2]=="RF01764"),27]<-"Orphan"

##average number of riboswitches per genome
b <- as.data.frame(gsub("_[0-9]*", "", a[,3]))
colnames(b) <- "Genome"
a2 <- cbind(b, a)
marine <- data.frame(a2[,1], a2[,2], a2[,28], Source="Marine")
colnames(marine) <- c("Genome", "Riboswitch", "Metabolite", "Source")

c3 <- data.frame(a2[,1], a2[,20], a2[,22], a2[,23], a2[,2], a2[,28],
Source="Marine")
colnames(c3) <- c("Genome", "Continent", "Phylum", "Class",
"Riboswitch", "Metabolite", "Source")

a<-read.table("Rfam_concatenated_oral.out")
a[which(a[,2]=="RF00059"),27]<-"Thiamine Pyrophosphate"
a[which(a[,2]=="RF00167"),27]<-"Purine"
a[which(a[,2]=="RF00050"),27]<-"Flavin Mononucleotide"
a[which(a[,2]=="RF03071"),27]<-"Orphan"
a[which(a[,2]=="RF01689"),27]<-"Cobalamin"
a[which(a[,2]=="RF00380"),27]<-"Magnesium"
a[which(a[,2]=="RF01055"),27]<-"Molybdenum Cofactor"
a[which(a[,2]=="RF01750"),27]<-"AICAR"
a[which(a[,2]=="RF03057"),27]<-"Orphan"
a[which(a[,2]=="RF01734"),27]<-"Fluoride"
a[which(a[,2]=="RF01725"),27]<-"S-adenosyl-methionine"
a[which(a[,2]=="RF00174"),27]<-"Cobalamin"
a[which(a[,2]=="RF00162"),27]<-"S-adenosyl-methionine"
a[which(a[,2]=="RF03072"),27]<-"Orphan"
a[which(a[,2]=="RF01739"),27]<-"Glutamine"
a[which(a[,2]=="RF00379"),27]<-"cyclic di-AMP"
a[which(a[,2]=="RF01831"),27]<-"Tetrahydrofolate"
a[which(a[,2]=="RF00442"),27]<-"Guanidine"
a[which(a[,2]=="RF02683"),27]<-"Nickel, Cobalt"
a[which(a[,2]=="RF01786"),27]<-"Cyclic di-GMP"
a[which(a[,2]=="RF01051"),27]<-"Cyclic di-GMP"
a[which(a[,2]=="RF03058"),27]<-"Orphan"
a[which(a[,2]=="RF01727"),27]<-"S-adenosyl-methionine"
a[which(a[,2]=="RF01057"),27]<-"S-adenosyl-L-homocysteine"
a[which(a[,2]=="RF00168"),27]<-"Lysine"
a[which(a[,2]=="RF00504"),27]<-"Glycine"
a[which(a[,2]=="RF00634"),27]<-"S-adenosyl-methionine"
```

```
a[which(a[,2]=="RF00521"),27]<-"S-adenosyl-methionine"
a[which(a[,2]=="RF00522"),27]<-"Pre-Queuosine"
a[which(a[,2]=="RF00080"),27]<-"Manganese"
a[which(a[,2]=="RF02680"),27]<-"Pre-Queuosine"
a[which(a[,2]=="RF01767"),27]<-"S-adenosyl-methionine"
a[which(a[,2]=="RF00234"),27]<-"Glucosamine-6-Phosphate"
a[which(a[,2]=="RF01054"),27]<-"Pre-Queuosine"
a[which(a[,2]=="RF01482"),27]<-"Cobalamin"
a[which(a[,2]=="RF02885"),27]<-"S-adenosyl-methionine"
a[which(a[,2]=="RF01826"),27]<-"S-adenosyl-methionine"
a[which(a[,2]=="RF02912"),27]<-"Aminoglycoside"
a[which(a[,2]=="RF01056"),27]<-"Magnesium"
a[which(a[,2]=="RF01510"),27]<-"Purine"
a[which(a[,2]=="RF01764"),27]<-"Orphan"
b <- as.data.frame(gsub("_[0-9]*", "", a[,3]))
colnames(b) <- "Genome"
a2 <- cbind(b, a)
oral <- data.frame(a2[,1], a2[,2], a2[,28], Source="Oral")
colnames(oral) <- c("Genome", "Riboswitch", "Metabolite", "Source")

c2 <- data.frame(a2[,1], a2[,20], a2[,22], a2[,23], a2[,2], a2[,28],
Source="Oral")
colnames(c2) <- c("Genome", "Continent", "Phylum", "Class",
"Riboswitch", "Metabolite", "Source")


a<-read.table("Human_Rfam_concat_cleaned.tblout")
a[which(a[,2]=="RF00059"),27]<-"Thiamine Pyrophosphate"
a[which(a[,2]=="RF00167"),27]<-"Purine"
a[which(a[,2]=="RF00050"),27]<-"Flavin Mononucleotide"
a[which(a[,2]=="RF03071"),27]<-"Orphan"
a[which(a[,2]=="RF01689"),27]<-"Cobalamin"
a[which(a[,2]=="RF00380"),27]<-"Magnesium"
a[which(a[,2]=="RF01055"),27]<-"Molybdenum Cofactor"
a[which(a[,2]=="RF01750"),27]<-"AICAR"
a[which(a[,2]=="RF03057"),27]<-"Orphan"
a[which(a[,2]=="RF01734"),27]<-"Fluoride"
a[which(a[,2]=="RF01725"),27]<-"S-adenosyl-methionine"
a[which(a[,2]=="RF00174"),27]<-"Cobalamin"
a[which(a[,2]=="RF00162"),27]<-"S-adenosyl-methionine"
a[which(a[,2]=="RF03072"),27]<-"Orphan"
a[which(a[,2]=="RF01739"),27]<-"Glutamine"
a[which(a[,2]=="RF00379"),27]<-"cyclic di-AMP"
a[which(a[,2]=="RF01831"),27]<-"Tetrahydrofolate"
a[which(a[,2]=="RF00442"),27]<-"Guanidine"
```

```
a[which(a[,2]=="RF02683"),27]<-"Nickel, Cobalt"
a[which(a[,2]=="RF01786"),27]<-"Cyclic di-GMP"
a[which(a[,2]=="RF01051"),27]<-"Cyclic di-GMP"
a[which(a[,2]=="RF03058"),27]<-"Orphan"
a[which(a[,2]=="RF01727"),27]<-"S-adenosyl-methionine"
a[which(a[,2]=="RF01057"),27]<-"S-adenosyl-L-homocysteine"
a[which(a[,2]=="RF00168"),27]<-"Lysine"
a[which(a[,2]=="RF00504"),27]<-"Glycine"
a[which(a[,2]=="RF00634"),27]<-"S-adenosyl-methionine"
a[which(a[,2]=="RF00521"),27]<-"S-adenosyl-methionine"
a[which(a[,2]=="RF00522"),27]<-"Pre-Queuosine"
a[which(a[,2]=="RF00080"),27]<-"Manganese"
a[which(a[,2]=="RF02680"),27]<-"Pre-Queuosine"
a[which(a[,2]=="RF01767"),27]<-"S-adenosyl-methionine"
a[which(a[,2]=="RF00234"),27]<-"Glucosamine-6-Phosphate"
a[which(a[,2]=="RF01054"),27]<-"Pre-Queuosine"
a[which(a[,2]=="RF01482"),27]<-"Cobalamin"
a[which(a[,2]=="RF02885"),27]<-"S-adenosyl-methionine"
a[which(a[,2]=="RF01826"),27]<-"S-adenosyl-methionine"
a[which(a[,2]=="RF02912"),27]<-"Aminoglycoside"
a[which(a[,2]=="RF01056"),27]<-"Magnesium"
a[which(a[,2]=="RF01510"),27]<-"Purine"
a[which(a[,2]=="RF01764"),27]<-"Orphan"
b <- as.data.frame(gsub("_[0-9]*", "", a[,3]))
colnames(b) <- "Genome"
a2 <- cbind(b, a)
human <- data.frame(a2[,1], a2[,2], a2[,28], Source="Human")
colnames(human) <- c("Genome", "Riboswitch", "Metabolite", "Source")

c <- data.frame(a2[,1], a2[,20], a2[,22], a2[,23], a2[,2], a2[,28],
Source="Gut")
colnames(c) <- c("Genome", "Continent", "Phylum", "Class",
"Riboswitch", "Metabolite", "Source")

a<-read.table("Mouse_Rfam_concat_cleaned.tblout")
a[which(a[,2]=="RF00059"),19]<-"Thiamine Pyrophosphate"
a[which(a[,2]=="RF00167"),19]<-"Purine"
a[which(a[,2]=="RF00050"),19]<-"Flavin Mononucleotide"
a[which(a[,2]=="RF03071"),19]<-"Orphan"
a[which(a[,2]=="RF01689"),19]<-"Cobalamin"
a[which(a[,2]=="RF00380"),19]<-"Magnesium"
a[which(a[,2]=="RF01055"),19]<-"Molybdenum Cofactor"
a[which(a[,2]=="RF01750"),19]<-"AICAR"
a[which(a[,2]=="RF03057"),19]<-"Orphan"
a[which(a[,2]=="RF01734"),19]<-"Fluoride"
```

```
a[which(a[,2]=="RF01725"),19]<-"S-adenosyl-methionine"
a[which(a[,2]=="RF00174"),19]<-"Cobalamin"
a[which(a[,2]=="RF00162"),19]<-"S-adenosyl-methionine"
a[which(a[,2]=="RF03072"),19]<-"Orphan"
a[which(a[,2]=="RF01739"),19]<-"Glutamine"
a[which(a[,2]=="RF00379"),19]<-"cyclic di-AMP"
a[which(a[,2]=="RF01831"),19]<-"Tetrahydrofolate"
a[which(a[,2]=="RF00442"),19]<-"Guanidine"
a[which(a[,2]=="RF02683"),19]<-"Nickel, Cobalt"
a[which(a[,2]=="RF01786"),19]<-"Cyclic di-GMP"
a[which(a[,2]=="RF01051"),19]<-"Cyclic di-GMP"
a[which(a[,2]=="RF03058"),19]<-"Orphan"
a[which(a[,2]=="RF01727"),19]<-"S-adenosyl-methionine"
a[which(a[,2]=="RF01057"),19]<-"S-adenosyl-L-homocysteine"
a[which(a[,2]=="RF00168"),19]<-"Lysine"
a[which(a[,2]=="RF00504"),19]<-"Glycine"
a[which(a[,2]=="RF00634"),19]<-"S-adenosyl-methionine"
a[which(a[,2]=="RF00521"),19]<-"S-adenosyl-methionine"
a[which(a[,2]=="RF00522"),19]<-"Pre-Queuosine"
a[which(a[,2]=="RF00080"),19]<-"Manganese"
a[which(a[,2]=="RF02680"),19]<-"Pre-Queuosine"
a[which(a[,2]=="RF01767"),19]<-"S-adenosyl-methionine"
a[which(a[,2]=="RF00234"),19]<-"Glucosamine-6-Phosphate"
a[which(a[,2]=="RF01054"),19]<-"Pre-Queuosine"
a[which(a[,2]=="RF01482"),19]<-"Cobalamin"
a[which(a[,2]=="RF02885"),19]<-"S-adenosyl-methionine"
a[which(a[,2]=="RF01826"),19]<-"S-adenosyl-methionine"
a[which(a[,2]=="RF02912"),19]<-"Aminoglycoside"
a[which(a[,2]=="RF01056"),19]<-"Magnesium"
a[which(a[,2]=="RF01510"),19]<-"Purine"
a[which(a[,2]=="RF01764"),19]<-"Orphan"
b <- as.data.frame(gsub("\\.[0-9]*", "", a[,3]))
colnames(b) <- "Genome"
a2 <- cbind(b, a)
mouse  <- data.frame(a2[,1], a2[,2], a2[,20], Source="Mouse")
colnames(mouse) <- c("Genome", "Riboswitch", "Metabolite", "Source")

c5 <- rbind(human, mouse, oral, marine)
c4 <- rbind(c, c2, c3)

#broad view of riboswitch classes
d <- c5 %>% group_by(Source) %>% count(Metabolite) %>%
arrange(desc(n)) %>% mutate( Metabolite = fct_reorder(Metabolite, n,
.desc=TRUE))
```

```
colourCount = length(unique(d$Metabolite))
getPalette = colorRampPalette(brewer.pal(8, "Set3"))

ggplot(d, aes(fill=Metabolite, y=n, x=Source)) +
geom_bar(position="fill", stat="identity") + scale_fill_manual(values
= getPalette(colourCount))

#rank order of human gut microbes
e<-d %>% filter(Source=="Human")%>% arrange(desc(n)) %>% mutate(
Metabolite = fct_reorder(Metabolite, n, .desc=TRUE))

colourCount = length(unique(e$Metabolite))
getPalette = colorRampPalette(brewer.pal(8, "Set3"))

ggplot(e, aes(y=n, x=Metabolite)) + geom_bar(stat="identity") +
scale_fill_manual(values = getPalette(colourCount)) +
theme(axis.text.x = element_text(angle = 90))

#average number of riboswitches per genome, broken down by class
d <- c %>% count(Genome, Class, sort = TRUE)
g <- d %>% group_by(Class) %>% summarise_at(vars(n), list(name =
mean)) %>% arrange(desc(name)) %>% mutate(Class = fct_reorder(Class,
name, .desc=TRUE))
ggplot(data = g, aes(x = Class, y = name)) + geom_bar(stat="identity")

#average number of riboswitches per genome, broken down by Phylum
d <- c %>% count(Genome, Phylum, sort = TRUE)
g <- d %>% mutate(Phylum = fct_reorder(Phylum, n, .desc=TRUE))
ggplot(g, aes(x=Phylum, y=n)) + geom_boxplot() + theme(axis.text.x =
element_text(angle = 45))

#breakdown of riboswitches by phylum
g <- c %>% group_by(Phylum) %>% count(Metabolite, .drop=FALSE) %>%
mutate( percent = n/sum(n))

g$ord.x <- factor(g$Metabolite, ordered=TRUE, levels =
c("Thiamine Pyrophosphate",
"Cobalamin",
"Flavin Mononucleotide",
"Lysine",
"S-adenosyl-methionine",
"Orphan",
"Glycine",
"Purine",
"AICAR",
```

```
"Tetrahydrofolate",
"Glucosamine-6-Phosphate",
"Fluoride",
"Magnesium",
"Molybdenum Cofactor",
"cyclic di-AMP",
"Molybdenum cofactor",
"Pre-Queuosine",
"Nickel, Cobalt",
"Guanidine",
"Manganese",
"Cyclic di-GMP",
"S-adenosyl-L-homocysteine",
"Aminoglycoside",
"Glutamine"))

#pdf("heatmap_distribution.pdf")
ggplot(data=g) + geom_tile(aes(x = ord.x, y = Phylum, fill = percent))
+ scale_fill_gradient(low = "white", high = "red") + theme(axis.text.x
= element_text(angle = 45))


ggplot(g, aes(fill=Metabolite, y=percent, x=Metabolite)) +
geom_bar(position="dodge", stat="identity") +
scale_fill_manual(values = getPalette(colourCount)) +
facet_wrap(~Phylum, dir="v")

ggplot(h, aes(y=percent, x=Phylum)) +
geom_bar(position="dodge", stat="identity") +
facet_wrap(~Metabolite, dir="v")

#list of riboswitches by phylum
h <- c %>% group_by(Metabolite) %>% count(Phylum) %>%
count(Metabolite) %>% arrange(desc(n))
print(h, n=nrow(h))

#pdf("Riboswitches_by_Phylum.pdf")
#ggplot(g, aes(fill=Metabolite, y=n, x=Phylum)) +
geom_bar(position="fill", stat="identity") + scale_fill_manual(values
= getPalette(colourCount)) + theme(axis.text.x = element_text(angle =
45))

#compare to oral microbiome
g <- c2 %>% group_by(Phylum) %>% count(Metabolite, .drop=FALSE) %>%
mutate( percent = n/sum(n))
```

```
ggplot(g, aes(fill=Metabolite, y=percent, x=Metabolite)) +
geom_bar(position="dodge", stat="identity") +
scale_fill_manual(values = getPalette(colourCount)) +
facet_wrap(~Phylum, dir="v")

#breakdown of riboswitches by continent
g <- c %>% filter(!is.na(Continent)) %>% group_by(Continent) %>%
count(Metabolite, .drop=FALSE) %>% mutate( Metabolite =
fct_reorder(Metabolite, n, .desc=TRUE))
#colourCount = length(unique(g$Riboswitch))
#getPalette = colorRampPalette(brewer.pal(11, "Set3"))
pdf("Riboswitch_Distribution_Continent.pdf")

ggplot(g, aes(fill=Metabolite, y=n, x=Continent)) +
geom_bar(position="fill", stat="identity") + scale_fill_manual(values
= getPalette(colourCount))

#breakdown of phyla by continent
g <- c %>% filter(!is.na(Continent)) %>% group_by(Continent) %>%
count(Phylum, .drop=FALSE)
ggplot(g, aes(fill=Phylum, y=n, x=Continent)) +
geom_bar(position="fill", stat="identity") +
scale_fill_manual(values = getPalette(colourCount))
pdf("Phylum_Distribution_Continent.pdf")

##>>Statistical testing
##>>For Actinobacter
h<- g %>% filter(Phylum == "p_Actinobacteriota")
r1 <- 132
r2 <- 45
h <- g %>% filter(Continent == "Africa")
r3 <- sum(h[,3])
h <- g %>% filter(Continent == "South_America")
r4 <- sum(h[,3])
prop.test(c(r1, r2), c(r3, r4), correct="F", alternative="two.sided")

##2-sample test for equality of proportions without continuity
correction
data:  c(r1, r2) out of c(r3, r4)
X-squared = 20.07, df = 1, p-value = 7.468e-06
alternative hypothesis: two.sided
95 percent confidence interval: 0.05296903 0.12697043
sample estimates:
    prop 1     prop 2
0.17837838 0.08840864
```

```
##>>Statistical testing
##>>For Firmicutes
h<- g %>% filter(Phylum == "p_Firmicutes")
r1 <- 503
r2 <- 405
h <- g %>% filter(Continent == "Africa")
r3 <- sum(h[,3])
h <- g %>% filter(Continent == "South_America")
r4 <- sum(h[,3])
prop.test(c(r1, r2), c(r3, r4), correct="F", alternative="two.sided")

2-sample test for equality of proportions without continuity
correction
data:  c(r1, r2) out of c(r3, r4)
X-squared = 20.427, df = 1, p-value = 6.196e-06
alternative hypothesis: two.sided
95 percent confidence interval: -0.16449767 -0.06739847
sample estimates:
 prop 1    prop 2
0.6797297 0.7956778

#specific riboswitches by continent
#none of the riboswitches were specific to any particular region
r1<-sum(mag[6,3][[1]])
r2<-sum(mag[2,3][[1]]+mag[3,3][[1]]+mag[4,3][[1]])
r3<-sum(geo[6,2][[1]])
r4<-sum(geo[2,2][[1]]+geo[3,2][[1]]+geo[4,2][[1]])

prop.test(c(r1, r2), c(r3, r4), correct="F", alternative="two.sided")

fl <- c %>% filter(!is.na(Continent)) %>% group_by(Continent) %>%
filter(Metabolite=="Fluoride") %>% count(Metabolite, sort = FALSE)

pur <- c %>% filter(!is.na(Continent)) %>% group_by(Continent) %>%
filter(Metabolite=="Purine") %>% count(Metabolite, sort = FALSE)

aminogly <- c %>% filter(!is.na(Continent)) %>% group_by(Continent)
%>%  filter(Metabolite=="Aminoglycoside") %>% count(Metabolite, sort =
FALSE)

diAMP <- c %>% filter(!is.na(Continent)) %>% group_by(Continent) %>%
filter(Metabolite=="cyclic di-AMP") %>% count(Metabolite, sort =
FALSE)
```

```
diGMP <- c %>% filter(!is.na(Continent)) %>% group_by(Continent) %>%
filter(Metabolite=="Cyclic di-GMP") %>% count(Metabolite, sort =
FALSE)

manga <- c %>% filter(!is.na(Continent)) %>% group_by(Continent) %>%
filter(Metabolite=="Manganese") %>% count(Metabolite, sort = FALSE)

mag <- c %>% filter(!is.na(Continent)) %>% group_by(Continent) %>%
filter(Metabolite=="Magnesium") %>% count(Metabolite, sort = FALSE)

molybd <- c %>% filter(!is.na(Continent)) %>% group_by(Continent) %>%
filter(Metabolite=="Molybdenum Cofactor") %>% count(Metabolite, sort =
FALSE)

glycine <- c %>% filter(!is.na(Continent)) %>% group_by(Continent) %>%
filter(Metabolite=="Glycine") %>% count(Metabolite, sort = FALSE)

geo <- c %>% filter(!is.na(Continent)) %>% group_by(Continent) %>%
count(Continent, sort = FALSE)

heo<-as.data.frame(cbind(fl[,3], diAMP[,3], manga[,3], mag[,3],
molybd[,3], glycine[,3], geo[,2]))

continent<-
c("Africa","Asia","Europe","North_America","Oceania","South_America","
NA")

concat<-cbind(continent, flN, diAN, mangaN, magN, molybN, glyN)
colnames(concat) <- c(continent, flN, diAN, mangaN, magN, molybN,
glyN)
```

Riboswitches that are over-represented in the human gut microbiome

```
#we can use the prop.test() command. This is also known as Z-test.
#prop.test() requires two inputs: a vector of #‘successes’ (numerator)
#and a vector of ‘counts’ (denominator).

#This is the table we want to query
t5 <- table(c5$Metabolite, c5$Source)
g <- nrow(t5)

c6 <- data.frame(matrix(ncol = 4, nrow = g))

#number of riboswitches of specific metabolite in non-gut microbiome
for (x in 1:g){
```

```
      #number of  riboswitches of specific metabolite in non-human
      r1 <- sum(t5[x,2], t5[x,3], t5[x,4])
      #r1 <- sum(t5[x,4])
      #number of  riboswitches of specific metabolite in human gut
      r2 <- sum(t5[x,1])
      #counts of non-human microbiome riboswitches
      r3 <- sum(colSums(t5)[2], colSums(t5)[3], colSums(t5)[4])
      #r3 <- sum(colSums(t5)[4])
      #counts of human gut microbiome riboswitches
      r4 <- sum(colSums(t5)[1])
      ztest <- prop.test(c(r1, r2), n = c(r3, r4), correct = F,
      alternative="two.sided")
      c6[x,1] <- ztest$estimate[1]
      c6[x,2] <- ztest$estimate[2]
      c6[x,3] <- ztest$estimate[2]/ztest$estimate[1]
      #--- Extract the z statistic
      c6[x,4] <- sqrt(ztest$statistic)
}
rownames(c6) <- rownames(table(c5$Metabolite, c5$Source))
colnames(c6) <- c("non-human","human_gut","ratio","Z.stat")
c6[ "class" ] <- rownames(c6)

c6 %>% mutate(class = fct_reorder(class, ratio)) %>%
  ggplot(aes(x=class, y=ratio)) +
  geom_bar(stat="identity", fill="skyblue", alpha=0.7) +
  theme(axis.text.x = element_text(angle = 45))

#how many of the comparisons had a ratio > 2.0
c6[which(c6$ratio > 2.5),]
```

Riboswitch distribution in individual genomes

```
#First cleanup the GFF file, to remove sequence data and headers
      ls -l *gff | awk '{print "grep \x22^MGYG\x22", $9, "> ",
      $9".tbl.cln.gff"}' | sed 's/.gff.tbl.cln.gff/.tbl.cln.gff/' >
      directions
      chmod +x directions
      ./directions
#Now you have a set of files with the gene annotations only

#Now cleanup the cmscan output so that you can adapt them to bedtools
      ls -l M*.tblout | awk '{print "perl ~/Programs/infernal-
      tblout2gff.pl --cmscan", $9, "> ",$9".cmscan.gff"}' | sed
      's/.tblout.cmscan.gff/.cmscan.gff/' > directions
      chmod +x directions
      ./directions
```

```
##Note that infernal-tblout2gff.pl comes from Eric Nawrockie at Sean
##Eddy's Lab
https://raw.githubusercontent.com/nawrockie/jiffy-infernal-hmmer-scripts/master/infernal-tblout2gff.pl

#Use bedtools to map the cmscan hits to the genome, use a 500nt
window, and must have same directionality (-sm option)
      ls -l *cmscan.gff | awk '{print "bedtools window -a", $9, "-b",
      $9".tbl.cln.gff -sm -l 0 -r 500 > "$9".cmscan.bed"}' | sed
      's/.cmscan.gff.tbl.cln.gff/.tbl.cln.gff/' | sed
      's/.cmscan.gff.cmscan.bed/.cmscan.bed/' > directions
      chmod +x directions
      ./directions
```

Determine the pathway functions associated with each riboswitch class

```
##For KEGG assignments
      grep -w "Cobalamin" all_riboswitch_locations.bed | awk
      'BEGIN{FS="KEGG="} {print $2}' | awk 'BEGIN{FS=";"} {print $1}' |
      sed '/-/d' | sed '/^$/d' | sed 's/ko://g' | sed 's/,/\n/g' | sort
      | uniq > cobalamin_KEGG_annotations.txt

#for standard product names
      grep -w "FMN" all_riboswitch_locations.bed | awk
      'BEGIN{FS="product="} {print $2}' | awk 'BEGIN{FS=";"} {print
      $1}' | sort | uniq -c | sort -rn
```

For this analysis, combined the following groups together:
B12: AdoCbl, AdoCbl_Variant, Cobalamin
c-di-GMP: class I and II
PreQ1: class I, II, and III
SAM: SAM, alpha, I-IV, IV, VI, SMK
Magnesium: ykoK, and Mg

To match KEGG orthology to pathways:
https://www.genome.jp/kegg/ko.html
Input the B12 KEGG annotations into KEGG "Map module"
Selected text and pasted into new document
Pasted a column with the counts and the module name in new txt files also

```
R
library(ggplot2)
a <-read.table("B12_KEGG_brite_mapper.txt")
colnames(a) <- c("Pathway", "Counts")
```

```
ggplot(a, aes(x = reorder(Pathway, -Counts), y = Counts)) +
geom_bar(stat = "identity") + coord_flip() + theme_bw(y.axis.text =
element_text(size = 5))
```

Find counts of riboswitches in gut microbiome

First, how many genes are there in this dataset?

>>Each GFF file has the list of all predicted CDS

```
ls -l *gff | awk '{print "grep -w \x22CDS\x22", $9, "| wc -l >>
gene_counts.txt"}' > directions
chmod +x directions
./directions
```

Then, if you look at the total number of riboswitches (actual hits in the genomic dataset) by counting the number of lines

```
wc -l Rfam_concat_cleaned.tblout
36844 Rfam_concat_cleaned.tblout
```

Patient level metagenomic samples

92 patient level metagenomic samples were downloaded from CNGB (Dataset 6 above)

Infernal was run on the samples as above

Then I performed a cleanup. The samples did not have the patient sample information embedded in the sequence information:

```
grep -v "#" HZ_Contigs.tblout | awk '{print "    HZ_   " ,$0}' >
HZ_infernal.clean.out
```

To create a shell script for all the samples I did the following, making three separate scripts I then stitched together:

```
ls -l *Contigs.tblout | awk '{print $9}' | sed 's/Contigs.tblout//' >
tmp
ls -l *tblout | awk '{print "grep -v \x22#\x22", $9, "| awk \x27{print
\x22"}' > tmp2
ls -l *tblout | awk '{print "\x22 ,$0 \x22}\x27 > ", $9}' | sed
's/Contigs.tblout/infernal.clean.out/' > tmp3
paste tmp2 tmp tmp3 > directions
Chmod +x directions
./directions
cat *infernal.clean.out > all_riboswitches_patient_samples.out

sed 's/raiA RNA/-/' all_riboswitches_patient_samples.out | sed
's/putative aminoglycoside riboswitch \/ attI site/-/' | sed 's/sul1
RNA/-/' | sed 's/yjdF RNA/-/' | sed 's/DUF1646 RNA/-/' | sed 's/nhaA-I
RNA/-/' | sed 's/Cyclic di-GMP-I riboswitch/-/' > tmp
mv tmp all_riboswitches_patient_samples.out
```

```
R
library(dplyr)
library(ggplot2)
library(forcats)
a <- read.table("all_riboswitches_patient_samples.out")
a[which(a[,3]=="RF00059"),20]<-"Thiamine Pyrophosphate"
a[which(a[,3]=="RF00167"),20]<-"Purine"
a[which(a[,3]=="RF00050"),20]<-"Flavin Mononucleotide"
a[which(a[,3]=="RF03071"),20]<-"Orphan"
a[which(a[,3]=="RF01689"),20]<-"Cobalamin"
a[which(a[,3]=="RF00380"),20]<-"Magnesium"
a[which(a[,3]=="RF01055"),20]<-"Molybdenum Cofactor"
a[which(a[,3]=="RF01750"),20]<-"AICAR"
a[which(a[,3]=="RF03057"),20]<-"Orphan"
a[which(a[,3]=="RF01734"),20]<-"Fluoride"
a[which(a[,3]=="RF01725"),20]<-"S-adenosyl-methionine"
a[which(a[,3]=="RF00174"),20]<-"Cobalamin"
a[which(a[,3]=="RF00162"),20]<-"S-adenosyl-methionine"
a[which(a[,3]=="RF03072"),20]<-"Orphan"
a[which(a[,3]=="RF01739"),20]<-"Glutamine"
a[which(a[,3]=="RF00379"),20]<-"cyclic di-AMP"
a[which(a[,3]=="RF01831"),20]<-"Tetrahydrofolate"
a[which(a[,3]=="RF00442"),20]<-"Guanidine"
a[which(a[,3]=="RF02683"),20]<-"Nickel, Cobalt"
a[which(a[,3]=="RF01786"),20]<-"Cyclic di-GMP"
a[which(a[,3]=="RF01051"),20]<-"Cyclic di-GMP"
a[which(a[,3]=="RF03058"),20]<-"Orphan"
a[which(a[,3]=="RF01727"),20]<-"S-adenosyl-methionine"
a[which(a[,3]=="RF01057"),20]<-"S-adenosyl-L-homocysteine"
a[which(a[,3]=="RF00168"),20]<-"Lysine"
a[which(a[,3]=="RF00504"),20]<-"Glycine"
a[which(a[,3]=="RF00634"),20]<-"S-adenosyl-methionine"
a[which(a[,3]=="RF00521"),20]<-"S-adenosyl-methionine"
a[which(a[,3]=="RF00522"),20]<-"Pre-Queuosine"
a[which(a[,3]=="RF00080"),20]<-"Manganese"
a[which(a[,3]=="RF02680"),20]<-"Pre-Queuosine"
a[which(a[,3]=="RF01767"),20]<-"S-adenosyl-methionine"
a[which(a[,3]=="RF00234"),20]<-"Glucosamine-6-Phosphate"
a[which(a[,3]=="RF01054"),20]<-"Pre-Queuosine"
a[which(a[,3]=="RF01482"),20]<-"Cobalamin"
a[which(a[,3]=="RF02885"),20]<-"S-adenosyl-methionine"
a[which(a[,3]=="RF01826"),20]<-"S-adenosyl-methionine"
a[which(a[,3]=="RF02912"),20]<-"Aminoglycoside"
a[which(a[,3]=="RF01056"),20]<-"Magnesium"
```

```
a[which(a[,3]=="RF01510"),20]<-"Purine"
a[which(a[,3]=="RF01764"),20]<-"Orphan"
b <- data.frame(a[,1], a[,3], a[,20])
colnames(b) <- c("Patient", "Rfam", "Metabolite")
d <- b %>% group_by(Patient) %>% count(Metabolite) %>%
arrange(desc(n)) %>% mutate( Metabolite = fct_reorder(Metabolite, n,
.desc=TRUE))

g <- d %>% group_by(Metabolite) %>% summarise(min(n), max(n), mean(n),
sd(n), n=n()) %>% arrange(desc(n))

   Metabolite                  `min(n)` `max(n)` `mean(n)` `sd(n)`     n
 1 Thiamine Pyrophosphate             4       37     12.6     5.73     92
 2 Cobalamin                          3       51     16.9    11.0      92
 3 S-adenosyl-methionine              1       18      7.53    3.68     90
 4 AICAR                              1       10      3.86    2.18     87
 5 Flavin Mononucleotide              1       14      4.49    2.91     85
 6 Glycine                            1       12      4.47    2.33     85
 7 Glucosamine-6-Phosphate            1        8      2.4     1.56     80
 8 Tetrahydrofolate                   1       10      2.68    1.73     75
 9 Lysine                             1       10      3.11    2.26     70
10 Cyclic di-GMP                      1       11      2.87    2.01     69
11 Fluoride                           1        6      1.81    1.12     69
12 Orphan                             1        5      1.70    0.905    67
13 Magnesium                          1        6      2.17    1.36     42
14 Purine                             1        6      1.97    1.44     39
15 Molybdenum Cofactor                1        3      1.49    0.651    37
16 Pre-Queuosine                      1        4      1.45    0.736    29
17 cyclic di-AMP                      1        2      1.08    0.289    12
18 Nickel, Cobalt                     1        2      1.22    0.441     9
19 Aminoglycoside                     1        1      1       0          7
20 Guanidine                          1        2      1.33    0.577      3
21 S-adenosyl-L-homocysteine          1        2      1.33    0.577      3
22 Manganese                          1        1      1       0          2
```
